# Role of SMC1A overexpression as a predictor of poor prognosis in late stage colorectal cancer

**DOI:** 10.1186/s12885-015-1085-4

**Published:** 2015-03-04

**Authors:** Jianwei Wang, Shaojun Yu, Liming Cui, Wenhui Wang, Jun Li, Ke Wang, Xinyuan Lao

**Affiliations:** 1 Department of Surgical Oncology, Second Affiliated Hospital, Zhejiang University School of Medicine, Hangzhou, 310009 China; 2 Holly Lab Shanghai, Shanghai, 200233 China

**Keywords:** Colorectal cancer, SMC1A, shRNA, Cell cycle, Prognosis

## Abstract

**Background:**

Structural maintenance of chromosomes 1A (SMC1A) is a member of the cohesion family of proteins that plays crucial roles in cell cycle control. Recent studies have concluded that SMC1A is involved in the pathogenesis of cancer. This study aims to evaluate the functional role of SMC1A in colorectal cancer (CRC) both in vitro and in vivo, and the underlying molecular mechanisms.

**Methods:**

We firstly investigated the expression levels of SMC1A in 427 CRC specimens. Antigen expression was determined by immunohistochemical analysis of SMC1A on tissue microarrays. Stable SMC1A knockdown CRC cell lines were employed. The effects of SMC1A depletion on cell growth in vitro were examined by MTT, colony formation and flow cytometry assays. Tumor forming was evaluated by nude mice model in vivo. To detect the activation of intracellular signaling, pathscan intracellular signaling array and western blotting were performed.

**Results:**

The expression of SMC1A was much stronger in CRC tumor tissues than in adenomas and normal colorectal tissues. High SMC1A expression, indicated as an independent poor prognostic predictor for patients with stage III and stage IV CRC, was correlated with overall survival (OS) (p = 0.008). Functional analysis indicated that SMC1A knockdown by small interfering RNA (siRNA) mediated the significant inhibition of cell proliferation; induced cell cycle arrest and apoptosis via the suppression of CDK4, PCNA and PARP; and blocked the activation of the Erk1/2 and Akt cascades in CRC cells. In addition, SMC1A depletion significantly decreased the growth of subcutaneously inoculated tumors in nude mice.

**Conclusions:**

These results suggest that SMC1A plays an essential role in the development of CRC and may be a predictive factor in patients with CRC. The inhibition of SMC1A may serve as a promising therapeutic strategy for human CRC.

## Background

Colorectal cancer (CRC) is the second leading cause of malignancy-related death after lung cancer [1]. If diagnosed at the early stages, CRC can be cured with surgery [2]. However, in most instances, the cancer has progressed to the malignant stage at the time of diagnosis [3]. Moreover, the mortality is increasing due to cancer metastasis [4,5]. Therefore, novel therapeutic strategies for the treatment of CRC are being widely researched. The identification of biomarkers related to CRC development and progression is a novel aspect of cancer research. Several genetic and epigenetic factors have been found to be involved in the progression of CRC [6]. These factors alter the apoptosis process of the cancer cells and facilitate cell growth and survival over normal cells [7]. In this regard, studies using RNA interference (RNAi) technology to target oncogenes have become popular [8].

Cohesin is a multiunit complex containing four subunits: a pair of SMC (structural maintenance of chromosomes) proteins, SMC1A and SMC3, and two non-SMC proteins, RAD21/Scc1 and STAG/Scc3/Sa. It associates with chromatin after mitosis and is important for holding sister chromatids together following DNA replication [9]. Moreover, the cohesion complex is required to regulate DNA damage-induced intra-S phase and G_2_/M checkpoints in mammalian cells because it facilitates the recruitment of proteins involved in cell cycle checkpoints [10]. The SMC protein family comprises 6 prominent members (SMC1 to SMC6) with different functions and distinct biochemical activities [11]. SMC1 serves as a target for the ATM protein, which is responsible for controlling DNA replication and repair in human cells [12]. SMC1A is a conserved member of the cohesion complex from yeast to humans, which has important roles in maintaining genome stability [13]. Mutation and deregulation of SMC1A are highly relevant to diverse human diseases, including Cornelia de Lange syndrome and malignant carcinomas. Narayan et al. demonstrated an upregulation of SMC1A mRNA in cervix cancer cells compared to normal cervix [14]. Recently, SMC1A was found to be associated with cell growth and survival in lung adenocarcinoma [15] and glioblastoma [16,17]. However, little is known concerning the possible role of SMC1A in human CRC.

Mutations of SMC1 have been identified in colorectal cancers [18]. Therefore, this study was designed to identify the relationship between SMC1A expression and CRC development. Additionally, the potential use of RNAi-mediated SMC1A gene knockdown as a therapeutic target against colon cancer progression was analyzed using in vitro and in vivo colon cancer cell models.

## Methods

### Patients and cell lines

The study population comprised three groups. Group A included 56 patients with normal rectal mucosa. Samples were obtained from patients with severe mixed hemorrhoids who underwent the Procedure for Prolapse and Hemorrhoids. All patients had morphologically normal colorectal mucosa that was free of neoplastic or inflammatory disease as confirmed by preoperative colonoscopy. Group B included 51 patients with colorectal adenomatous polyps. All these polyps were resected endoscopically and proven to be adenomas by postoperative pathological examination. Group C included 427 patients with sporadic CRC, including 53 patients with stage I disease, 159 with stage II disease, 160 with stage III disease and 55 with stage IV disease. Each patient had an available specimen of a resected primary CRC. All patients with CRC were classified according to the TNM staging system using the International Union Against Cancer criteria. Patients who were diagnosed with cancers of any other histotype and those with a family history of CRC were excluded from the study. We enrolled 427 CRC patients with a median age of 60 years (range, 20–87 years), and 240 of these patients were male. All 427 patients did not receive preoperative chemotherapy or radiotherapy. However, patients with stage III and stage IV disease received 5-fluorouracil-based systemic chemotherapy after surgery. Postoperative adjuvant radiation was also administered to those patients with stage III and stage IV CRC. Patients were followed until death or December 18, 2011, with a mean postoperative follow-up duration of 40 months. Disease-free survival (DFS) was defined as the duration from the date of surgery to the date of first confirmed disease recurrence or to the date of the last follow-up for those without disease recurrence. All patients underwent surgery in the Department of Colorectal Surgery, The Second Affiliated Hospital, Medical School of Zhejiang University, China between April 2001 and December 2009. All patients provided informed consent. This study was approved by the Committee on Ethics of Biomedical Research, The Second Affiliated Hospital, Medical School of Zhejiang University, China.

Human CRC cell lines were obtained from Chinese Academy of Sciences Cell Bank of Type Culture Collection (SW480: #TCHu172, SW620: #TCHu101, RKO: #TCHu116, DLD-1: #TCHu134, HCT 116: #TCHu 99) and maintained in RPMI1640 (Hyclone) supplemented with 10% heat-inactivated FBS at 37°C in a humidified atmosphere of 5% CO_2_. Human embryonic kidney cell line (HEK-293: #GNHu43) was maintained in DMEM (Hyclone) supplemented with 10% heat-inactivated FBS and penicillin/streptomycin.

### Tissue microarray and immunohistochemistry (IHC)

All CRC cases were histologically reviewed by hematoxylin and eosin staining, and representative areas were premarked in paraffin blocks, away from necrotic and hemorrhagic materials. Cylinders measuring 1.5 mm in diameter were then taken from the paraffin blocks. Finally, 4 different tissue microarray (TMA) blocks were constructed, containing a total of 534 cores (56 normal mucosa cores, 51 adenoma cores and 427 CRC cores). Sections of 4-μm thickness were placed on three aminopropyltriethoxysilane-coated slides.

Immunohistochemistry (IHC) was performed using a standard streptavidin-biotin-peroxidase complex method as described in our previous report [19]. Slides were incubated overnight at 4°C with anti-SMC1A (1:200, Santa Cruz Biotechnology, Inc) and the antibody specificity was validated according to a previous report [20]. SMC1A expression was estimated based on the percentage and intensity of the stained tumor cells. The staining percentage was graded as 0 (0-5%), 1 (6-20%), 2 (21-60%) and 3 (61-100%), and the staining intensity was graded as 0 (negative), 1 (weak), 2 (moderate) and 3 (strong). The sum of the intensity and extent score was used as the final staining score (0–6). Tumors with final staining scores of 0, 1, 2–4 and 5–6 were considered to be negative (−), slightly positive (+), moderately positive (++) and strongly positive (+++), respectively, as described previously [21,22].

### Construction of SMC1A shRNA containing lentivirus and infection into cancer cells

The short hairpin RNA (shRNA) for human SMC1A (GenBank: NM_006306) (5′-CTAGCCCGGGCCGGGACTGTATTCAGTATACTCGAGTATACTGAATACAGTCCCGGCTTTTTTGTTAAT-3′) was screened and validated to be a candidate shRNA. A non-silencing siRNA (5′-TTCTCCGAACGTGTCACGT-3′) was used as control. To eliminate the possible off-target effects of shRNA, another shRNA against SMC1A (5′- TAACAAAAAAGCCGGGACTGTATTCAGTATACTCGAGTATACTGAATACAGTCCCGGCCCGGG-3′) was used to obtain comparable results. The oligos were inserted into the pFH-L vector (Holly Lab Shanghai). The lentiviral particles were constructed according to previous report [23]. RKO and SW480 cells were infected with lentivirus containing SMC1A shRNA (Lv-shSMC1A) or control shRNA (Lv-shCon) at an MOI of 30 or 60, respectively. The cells (50,000 cells/well) were seeded in 6-well plates, and successful infection was confirmed after 72 h by observation through a fluorescence microscope for green fluorescence protein expression.

### Real-time quantitative PCR analysis

RT-PCR analysis was performed using the SYBR Green Master Mix Kit in the DNA Engine Opticon™ System (MJ Research, Waltham, MA) as described in previous reports [24]. Beta-actin was used as an internal control. The primers of SMC1A were 5′-GCAGCAGCAGCAGATTGAG-3′ (forward) and 5′-TCTCTTCTTCCATCCGTTCTTC-3′ (reverse). The primers of β-actin were 5′-GTGGACATCCGCAAAGAC-3′ (forward) and 5′-AAAGGGTGTAACGCAACTA-3′ (reverse). The relative gene expression levels were calculated and statistically compared using the 2^-ΔΔCT^ analysis program.

### MTT assay

After lentivirus infection, RKO and SW480 cells were seeded at 1.2 × 10^3^ cells/well and 2 × 10^3^ cells/well, respectively, into 96-well plates. Cell viability was analyzed using the MTT assay. Briefly, 20 μl of the MTT solution (5 mg/ml) was added to each well after 1–5 days. The samples were incubated at 37°C for 4 h, and then dissolved in 100 μl of acidic isopropanol (10% SDS, 5% isopropanol and 0.01 mol/L HCl). The optical density was measured using a microplate reader at 595 nm.

### Colony formation assay

Lentivirus-transduced cells (200 cells/well) were seeded into 6-well plates and the medium was replaced every three days. After 8 days of incubation, cells were washed with PBS and fixed with 4% paraformaldehyde for 30 min at room temperature. The fixed cells were stained with Giemsa (Merck) for 20 min, washed with water and air-dried. The total number of colonies with more than 50 cells was counted using a light microscope and a fluorescence microscope.

### Flow cytometry analysis

After lentivirus infection, RKO cells (1 × 10^5^ cells/dish) were seeded on 6-cm dishes and collected until 80% confluence. The cells were then fixed by suspension in 0.7 ml of 70% ethanol for 30 min at 4°C. The ethanol was discarded after centrifugation, and a propidium iodide (PI, 100 μg/ml) solution containing 10 μg/ml of DNase-free RNase A was added to stain the cells, followed by incubation for 30 min at room temperature. The cell suspension was next filtered through a 50-μm nylon mesh, and 10,000 stained cells were analyzed using flow cytometer (Cell Lab Quanta, Beckman Coulter).

Apoptosis was detected by Annexin V-APC/7-AAD apoptosis detection kit (KeyGEN Biotech, Nanjing, China) following manufacturer’s instructions.

### Tumorigenesis in nude mice

Five- to six-week-old nude mice were inoculated with RKO cells. Prior to inoculation, three groups of RKO cells were prepared: Lv-shSMC1A-infected RKO cells, Lv-shCon-infected RKO cells and non-infected RKO cells. Twenty-four mice were randomized into three groups with eight mice in each group. Next, after the passage of three generations, the cells were suspended in physiological saline solution at a cell density of 5 × 10^7^ cells/ml. From the cell suspension, 0.2 ml was injected subcutaneously into the mice using a 6-gauge, 1-ml needle. The mice were reared until the tumor was visible to the naked eye. The measurements of the tumor (diameter and size) were measured at 6, 9, 12, 15 and 20 days after inoculation. The mice were euthanized after 20 days of inoculation, and then the size and weight of the colon tumors were measured and compared among the three groups. All animal treatments were performed strictly in accordance with international ethical guidelines and the National Institutes of Health Guide concerning the Care and Use of Laboratory Animals. The experiments were approved by the Institutional Animal Care and Use Committee of Zhejiang University.

### Path Scan intracellular signaling array and Western blot analysis

To detect the activation of intracellular signaling, the PathScan intracellular signaling array was used. Briefly, 5 days after lentivirus infection, RKO cells were collected and lysed. Intracellular signaling was detected using a PathScan intracellular signaling array kit (Cell Signaling Technology) following the manufacturer’s instructions.

Western blot was performed according to standard protocols, as described previously [25]. The primary antibodies used in this study were as follows: anti-SMC1A (1:200, Santa Cruz Biotechnology, Inc); anti-GAPDH (1:3000, Santa Cruz Biotechnology, Inc); anti-Erk (1:2000, Santa Cruz Biotechnology, Inc); anti-phospho-Erk1/2 (1:2000, Signalway Antibody); anti-PCNA (1:1500, MBL); anti-PARP and anti-CDK4 (1:1000, all from Cell Signaling Technology).

### Statistical analysis

Associations between SMC1A expression and clinicopathological variables were analyzed by Pearson Chi-Square test. The median OS and DFS, as well as their 95% confidence intervals (95% CIs), were estimated by the Kaplan-Meier method. The difference in survival was analyzed using the log-rank test. Student’s *t*-test was used to evaluate the differences between the SMC1A silenced and non-silenced groups. A p value less than 0.05 (two-sided) was considered to be statistically significant. All statistical analyses were conducted using SPSS17.0 statistical software (SPSS Inc, Chicago, IL).

## Results

### SMC1A expression in normal mucosa, adenoma and stage I to IV CRC

**Figure 1:**
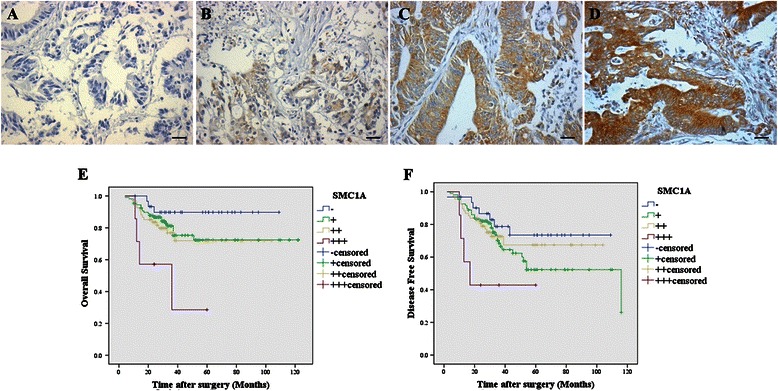
**SMC1A staining is demonstrated mainly at the membrane of tumor cells. Positive cells were stained brown.** The intensity of SMC1A staining was assigned the following scores: negative = − **(A)**, slightly positive = + **(B)**, moderately positive = ++ **(C)** and strongly positive = +++ **(D)**. Examples of representative immunohistochemistry results are shown. Scale bars = 50 μm. Kaplan-Meier analysis of **(E)** overall survival (p = 0.008) and **(F)** disease-free survival (p = 0.087) for SMC1A expression in patients with high-grade (stage III and stage IV) CRC (n = 215) is shown.

The expression of SMC1A in normal mucosa, adenoma and stage I to IV CRC is presented in Table 1. Strong SMC1A expression in CRC tissues was significantly higher than that in matched adenoma and normal mucosa. There were significant differences noted regarding SMC1A expression among these three groups (p = 0.015). Representative photomicrographs of four degrees of SMC1A expression intensity are shown in Figure 1A-D.

**Table 1:** **Expression of SMC1A in normal mucosa, adenoma and stage I to IV colorectal cancer (n = 534)**

Characteristic	Total	SMC1A immunostaining				P^a^
		-	+	++	+++	
Normal mucosa	53	6 (11.3%)	32 (60.4%)	14 (26.4%)	1 (1.9%)	0.015
Adenoma	50	10 (20.0%)	31 (62.0%)	9 (18.0%)	0 (0.0%)	
Colorectal cancer (Stage I-IV)	426	41 (9.6%)	207 (48.6%)	162 (38.0%)	16 (3.8%)	

^a^using Pearson Chi-Square test. 5 cases are missing (0.9%).

### Correlation between SMC1A expression and clinicopathologic parameters of patients with CRC

The association between SMC1A expression and the clinicopathologic parameters is shown in Table 2. Higher expression of SMC1A was found to be significantly associated with distant metastasis (p = 0.022) and higher TNM stage of disease (p < 0.001). In addition, SMC1A expression in colon cancer was much higher than that in rectal cancer (p < 0.001). No significant association was observed between SMC1A expression and patient gender, patient age, invasion depth, tumor differentiation, serum CEA level, serum CA19-9 level or recurrence.

**Table 2:** **Relationship of SMC1A expression and clinicopathological parameters in colorectal cancer patients (n = 427)**

Characteristic	N	SMC1A immunostaining				P^a^
		-	+	++	+++	
Gender (n)						0.067
Male	240	21 (8.8%)	128 (53.3%)	80 (33.3%)	11 (4.6%)	
Female	186	20 (10.8%)	79 (42.5%)	82 (44.1%)	5 (2.7%)	
Age (years)						0.193
≤60	227	27 (11.9%)	112 (49.3%)	82 (36.1%)	6 (2.6%)	
>60	199	14 (7.0%)	95 (47.7%)	80 (40.2%)	10 (5.0%)	
Position						<0.0001
Colon cancer	209	6 (2.9%)	99 (47.4%)	93 (44.5%)	11 (5.3%)	
Rectal cancer	217	35 (16.1%)	108 (49.8%)	69 (31.8%)	5 (2.3%)	
Invasion depth						0.388
T1-T2	79	11 (13.9%)	34 (43.0%)	32 (40.5%)	2 (2.5%)	
T3-T4	347	30 (8.6%)	173 (49.9%)	130 (37.5%)	14 (4.0%)	
Lymph node metastasis						0.001
N0	229	10 (4.4%)	107 (46.7%)	103 (45.0%)	9 (3.9%)	
N1	131	19 (14.5%)	65 (49.6%)	41 (31.3%)	6 (4.6%)	
N2	66	12 (18.2%)	35 (53.0%)	18 (27.3%)	1 (1.5%)	
Distant metastasis						0.022
M0	372	40 (10.8%)	186 (50.0%)	133 (35.8%)	13 (3.5%)	
M1	54	1 (1.9%)	21 (38.9%)	29 (53.7%)	3 (5.6%)	
TNM						<0.0001
I	53	7 (13.2%)	22 (41.5%)	23 (43.4%)	1 (1.9%)	
II	159	3 (1.9%)	77 (48.4%)	71 (44.7%)	8 (5.0%)	
III	160	30 (18.8%)	87 (54.4%)	39 (24.4%)	4 (2.5%)	
IV	54	1 (1.9%)	21 (38.9%)	29 (53.7%)	3 (5.6%)	
Tumor differentiation						0.161
Well	13	1 (7.7%)	7 (53.8%)	5 (38.5%)	0 (0.0%)	
Moderately	358	30 (8.4%)	170 (47.5%)	143 (39.9%)	15 (4.2%)	
Poorly	55	10 (18.2)	30 (54.5%)	14 (25.5%)	1 (1.8%)	
Serum CEA						0.190
≤5 ng/mL	280	21 (7.5%)	137 (48.9%)	112 (40.0%)	10 (3.6%)	
>5 ng/mL	146	20 (13.7%)	70 (47.9%)	50 (34.2%)	6 (4.1%)	
Serum CA199						0.776
≤37 U/ml	358	31 (8.7%)	176 (49.2%)	138 (38.5%)	13 (3.6%)	
>37 U/ml	65	8 (12.3%)	31 (47.7%)	23 (35.4%)	3 (4.6%)	
Recurrence						0.536
No	338	34 (10.1%)	161 (47.6%)	132 (39.1%)	11 (3.3%)	
Yes	88	7 (8.0%)	46 (52.3%)	30 (34.1%)	5 (5.7%)	

^a^using Pearson Chi-Square test. 1 case is missing (0.2%).

### Correlation between SMC1A expression and survival in patients with CRC

In late stage (stage III and stage IV) CRC cases, a higher SMC1A expression was found to be significantly associated with worse OS (p = 0.008) (Figure 1E). The OS of those patients with negative SMC1A expression (−) was significantly higher than that of patients with strong positive SMC1A expression (+++). The DFS of patients with negative SMC1A expression (−) was also obviously higher than that of patients with strong positive SMC1A expression (+++) (Figure 1F).

### shRNA-mediated SMC1A knockdown efficiency in CRC cells

**Figure 2:**
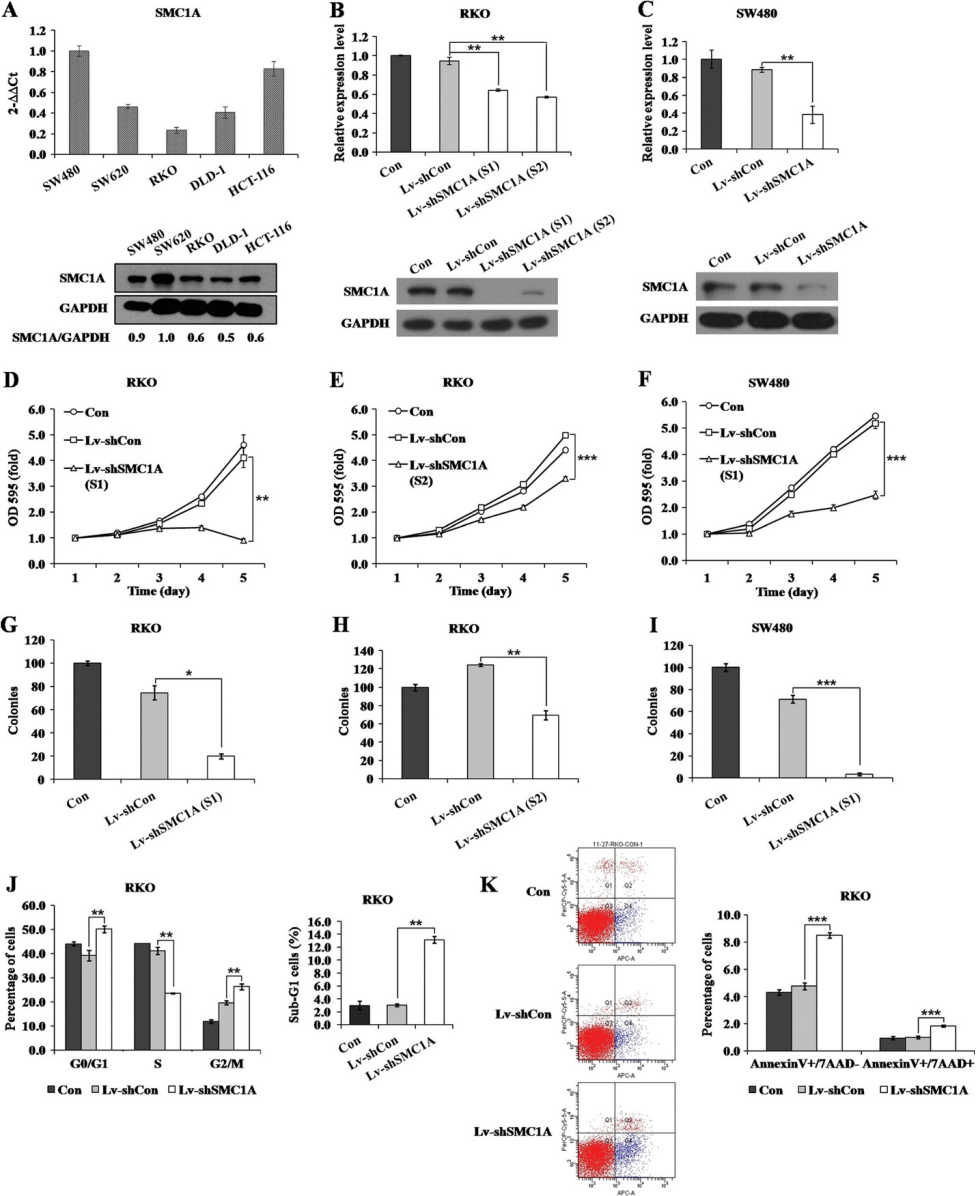
**Functional analysis of SMC1A by shRNA in CRC cells. (A)** Expression analysis of SMC1A in five different CRC cell lines by real-time PCR (upper panel) and western blotting (lower panel). **(B)** Expression analysis of SMC1A in RKO cells after Lv-shSMC1A infection by real-time PCR (upper panel) and western blotting (lower panel). **(C)** Expression analysis of SMC1A in SW480 cells after Lv-shSMC1A infection by real-time PCR (upper panel) and western blotting (lower panel). β-actin gene and GAPDH protein were used as internal controls. The proliferation levels of RKO **(D, E)** and SW480 **(F)** cells after Lv-shSMC1A infection analyzed by the MTT assay. The number of colonies in RKO **(G, H)** and SW480 **(I)** cells after Lv-shSMC1A infection analyzed by the colony formation assay. **(J)** The percentages of RKO cells using three different treatments in different phases (left panel) and the sub-G1 phase (right panel) of the cell cycle. **(K)** RKO cells stained with Annexin V and 7-AAD analyzed using flow cytometer (left panel). Q1, Annexin V^−^/7-AAD^+^; Q2, Annexin V^+^/7-AAD^+^; Q3, Annexin V^−^/7-AAD^−^; Q4, Annexin V^+^/7-AAD^−^. Quantification of the percentage of early apoptotic cells and late apoptotic cells (right panel). *p < 0.05, **p < 0.01, ***p < 0.001 in comparison with non-silencing shRNA infected control.

As shown in Figure 2A, the expression of SMC1A was observed in all five CRC cell lines. RKO and SW480 cell lines were used to investigate loss of function in the following study. Both cell lines were cultured and successfully infected with Lv-shCon or Lv-shSMC1A with an infection rate greater than 80%. The expression levels of SMC1A were significantly decreased (p < 0.01) in Lv-shSMC1A groups (Figure 2B and C). Similar result was observed in RKO cells treated with another shRNA against SMC1A. These results indicated that the lentivirus-mediated shRNA targeting SMC1A could effectively knock down SMC1A expression in CRC cells.

### Functional analysis of SMC1A by shRNA in CRC cells

As shown in Figure 2D and F, the proliferation rates of Lv-shSMC1A-infected cells started to decrease and were significantly reduced compared with Lv-shCon groups at the 4th and 5th days. Moreover, the number and size of the colonies were remarkably decreased in both RKO and SW480 cells after SMC1A knockdown (Figure 2G and I). Similar results were observed in RKO cells treated with another shRNA against SMC1A (Figure 2E and H). These results indicated that SMC1A could play an essential role in CRC cell proliferation and tumorigenesis in vitro.

To examine the mechanism underlying the inhibition of cell growth, the cell cycle distribution and apoptosis were detected in RKO cells after lentivirus infection. As shown in Figure 2J, compared with Lv-shCon groups, the percentages of cells in the G0/G1 and G2/M phases were significantly increased, whereas the number of cells in the S phase was significantly decreased in RKO cells infected with Lv-shSMC1A. Moreover, in the absence of SMC1A, more cells were obviously accumulated in the sub-G1 phase representing apoptotic cells. As shown in Figure 2K, Annexin V-APC vs 7-AAD plots from the gated cells showed the populations corresponding to viable and non-apoptotic (Annexin V^−^/7-AAD^−^), early (Annexin V^+^/7-AAD^−^), and late (Annexin V^+^/7-AAD^+^) apoptotic cells. After Lv-shSMC1A infection, more cells were Annexin V positive and 7-AAD negative, which represents early apoptosis. These results indicated that the cell cycle was arrested in the G0/G1 and G2/M phases and that RKO cells were entering early apoptotic stage after SMC1A knockdown.

### Effect of SMC1A knockdown on tumorigenesis in vivo

**Figure 3:**
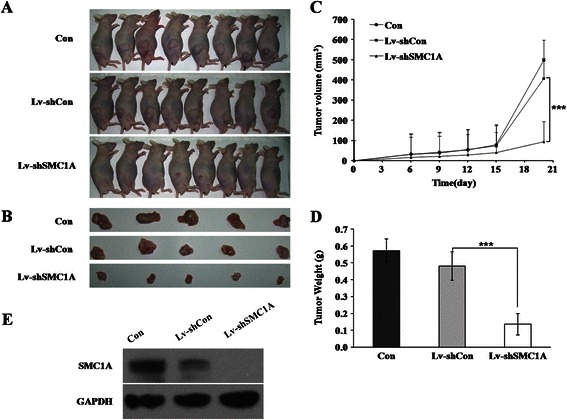
**Effect of SMC1A silencing on the tumorigenesis of CRC in vivo. (A)** The subcutaneous xenograft murine model. **(B)** Representative images of tumors formed in the mice in which Lv-shSMC1A- or Lv-shCon-infected RKO cells were implanted. **(C)** Changes in the tumor volume on the 6th, 9th, 12th, 15th and 21st days after the cells were implanted subcutaneously. **(D)** Changes in the tumor weight in mice after SMC1A silencing. **(E)** Expression analysis of SMC1A in tumor tissues collected from mice. ***p < 0.01 in comparison with non-silencing shRNA infected control.

The effects of SMC1A silencing on the development of colon tumors were analyzed in vivo using nude mouse models. As shown in Figure 3A and B, the size of the tumors was significantly decreased after SMC1A knockdown in a time course of 20 days. Moreover, the time-dependent analysis showed that the development of tumors peaked after 15 days, and the volume of the tumors was significantly suppressed in mice inoculated with SMC1A-silenced RKO cells compared with control groups (Figure 3C). Furthermore, the weight of the tumors was also remarkably decreased (p < 0.001) in SMC1A-silenced mice (Figure 3D). These results were confirmed by analysis of the protein content of SMC1A in tissues of a subcutaneous xenograft murine model. The SMC1A expression level was completely suppressed in tumor tissues by the infection with SMC1A shRNA (Figure 3E). This finding indicated that colon tumorigenesis was significantly inhibited by the absence of SMC1A.

### Mechanism study of SMC1A silencing in CRC cells

**Figure 4:**
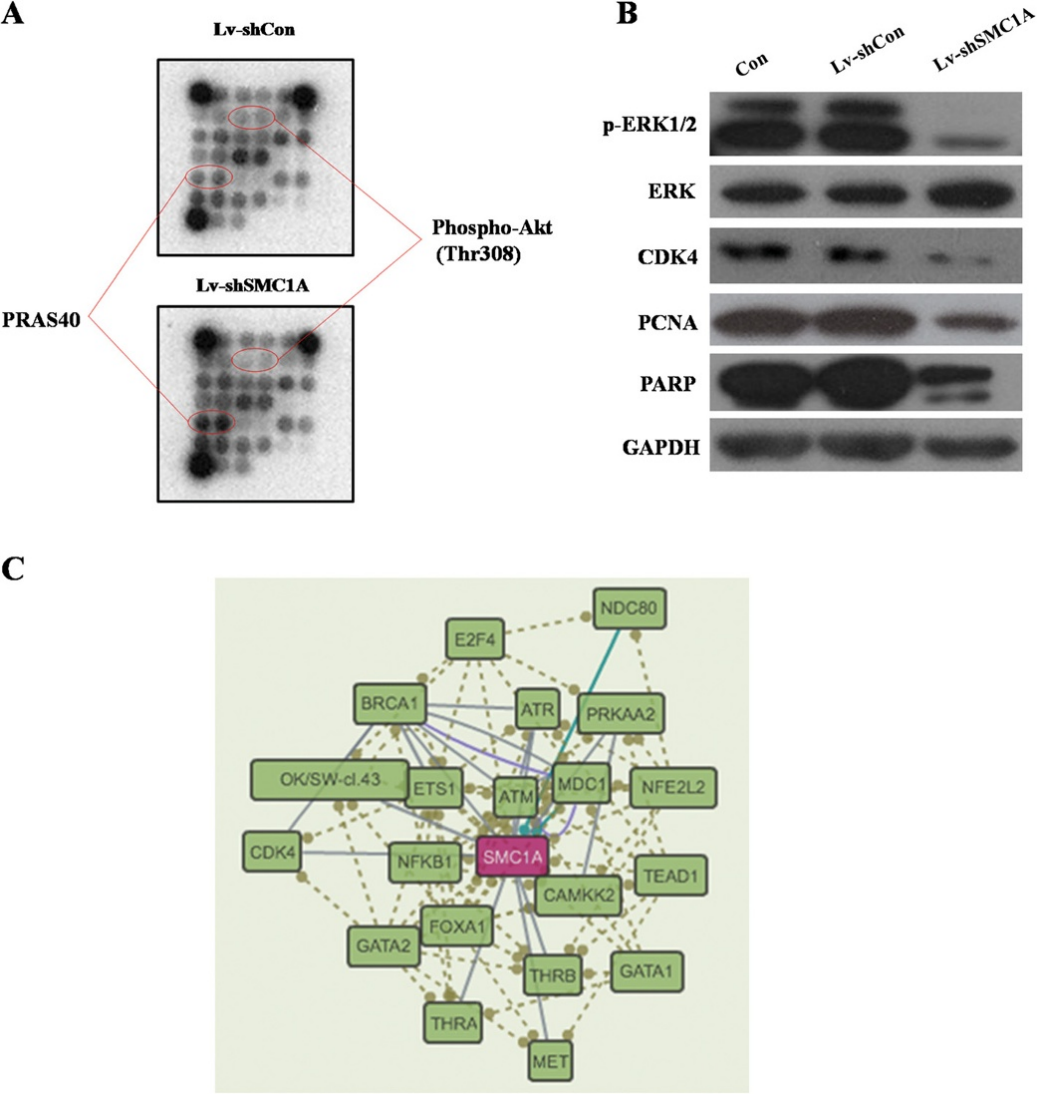
**Mechanism study of SMC1A silencing in CRC cells. (A)** Intracellular signaling array after Lv-shSMC1A infection. **(B)** Western blotting analysis of SMC1A-related signaling molecules in RKO cells. **(C)** SignaLink 2.0 analysis of SMC1A-related signaling molecules in RKO cells.

To investigate the regulatory mechanism of SMC1A in the tumorigenesis of CRC, multiple signaling pathways were analyzed in SW480 cells after SMC1A knockdown. SMC1A-triggered signal transduction was determined using the PathScan intracellular signaling array kit. Knockdown of SMC1A obviously inhibited the activation of Akt and induced the activation of PRAS40 (Figure 4A). Moreover, depletion of SMC1A significantly inhibited the phosphorylation of Erk1/2, indicating that SMC1A affected cell proliferation possibly via the tyrosine kinase-activated Ras/MEK/Erk pathway and Ras/PI3K/Akt pathway. In addition, SMC1A silencing decreased the expression levels of CDK4, PCNA and PARP in SW480 cells, indicating that CDK4, PCNA and PARP play important roles in SMC1A-induced cell cycle arrest and apoptosis (Figure 4B). Furthermore, a signaling pathway resource with multi-layered regulatory networks was applied to identify SMC1A-related signaling molecules (Figure 4C). Further studies are needed to clarify the mechanisms of SMC1A in CRC progression.

## Discussion

SMC1A is a member of the cohesion complex that is involved in critical cellular functions such as DNA repair, cell cycle progression, gene expression regulation and the maintenance of genome stability [11,26]. Additionally, dysfunction of the proteins in the cohesion complex was found to lead to genome instability, which is directly related to cancer development. In the present study, we found that SMC1A expression was significantly elevated in human CRC tissues. In addition, the increased expression of SMC1A was positively associated with worse clinicopathologic variables, including distant metastasis and higher TNM stage. Interestingly, the results of the current study demonstrated a significant correlation between SMC1A overexpression and shortened OS in patients with advanced CRC. Metastasis and a higher TNM stage are widely believed to be responsible for the worse prognosis noted among patients with CRC. These results suggest that patients with CRC with a higher expression of SMC1A in their tumors have a worse OS.

Previous studies have reported that RNAi-mediated gene silencing could be effectively used to suppress the proliferation of colon cancer cells in vitro [7,27]. In the present study, the CRC cell models and mouse models were shown to express elevated amounts of SMC1A. These findings suggested that the overexpression of SMC1A resulted in genome instability and led to the development and progression of CRC cells. shRNA-mediated SMC1A knockdown resulted in a significant down-regulation of cell proliferation, colony formation, cell cycle progression and a significant up-regulation of apoptosis in CRC cells. Our results were coincident with the outcome of SMC1A silencing in lung adenocarcinoma cells, in which cell growth was suppressed through G0/G1 phase cell cycle arrest and apoptosis [15]. Also, knocking down SMC1A inhibited growth and led to G2/M arrest in glioma cells [17]. From these data, we suggest that SMC1A plays an essential role in CRC cell growth. Moreover, the size of colon tumors was significantly decreased in the absence of the SMC1A gene, indicating that the up-regulated expression of SMC1A has a positive impact on the progression of CRC cells and that shRNA-mediated gene silencing effectively down-regulates CRC progression in both in vitro and in vivo models.

Intracellular signaling array showed that SMC1A depletion inhibited the activation of Akt and induced the activation of PRAS40. Phosphorylation of Akt at Ser473 and Thr308 by the TORC2 complex and PDK1, respectively, are reliable predictors of Akt activation. Phosphorylation of PRAS40 at Thr246 by Akt relieves the PRAS40 inhibition of TORC1 [28]. PRAS40 knockdown reduced the ability of tumor necrosis factor (TNF)-α and cycloheximide to induce apoptosis in HeLa cells [29]. Further analysis demonstrated that SMC1A silencing attenuates the activation of Erk1/2 and Akt, which are generally activated in response to growth factor stimulation that transmits growth and survival signals [30]. Thus, the mechanisms of SMC1A knockdown restricting CRC cell growth may occur, in part, via the blockade of Akt and MAP kinase activation. Cyclins and cdks are two types of crucial regulatory molecules that determine cell cycle progression [31]. Cyclin D1 binding to CDK4/6 forms the active complex of Cyclin D1-CDK4/6, which phosphorylates retinoblastoma protein (pRb) and subsequently releases E2F transcription factors, resulting in the activation of specific gene expression required for G1 to S phase progression [19]. Moreover, SMC1A was suggested to regulate CDK4 as analyzed by SignaLink 2 [32,33]. Cell cycle analysis showed that SMC1A knockdown restricted G1 to S phase progression, and further investigation demonstrated that SMC1A silencing down-regulated the expression of CDK4. PCNA is a member of the DNA sliding clamp family of proteins that assists in DNA replication and repair, and considered to be a marker of cell proliferation in various cancers [34]. In addition, PCNA also forms complexes with Cyclin-CDK complexes and acts as a connector between CDK and its substrates, stimulating their phosphorylation and thus controlling cell cycle progression [35,36]. Poly-ADP-ribose polymerase (PARP), a member of the PARP enzyme family, is an abundant DNA-binding enzyme that detects and signals DNA strand breaks [37]. The presence of cleaved PARP-1 is one of the most used diagnostic tools for the detection of apoptosis in many cell types [38]. Our results revealed that SMC1A silencing also down-regulates PCNA and PARP expression. Collectively, the mechanisms of SMC1A knockdown inhibiting CRC cell proliferation and cell cycle progression may occur, in part, via the blockade of Akt and MAP kinase activation and the subsequent suppression of CDK4 and PCNA. Knockdown of SMC1A induced apoptosis, potentially due to the induction of PRAS40 and the cleavage of PARP.

## Conclusions

SMC1A may be a predictive factor in patients with CRC, in whom high SMC1A expression is predictive of a poor prognosis. In the absence of SMC1A, cell proliferation, cell cycle progression and tumor development were effectively suppressed. Therefore, shRNA-mediated SM1CA silencing could be an effective therapeutic tool for colon cancer treatment. We propose that the overexpression of SMC1A may lead to CRC development by inducing cell growth and inhibiting apoptosis. However, determining how SMC1A is relevant to the etiology of CRC requires further investigation.
